# Mutation in the Pro-Peptide Region of a Cysteine Protease Leads to Altered Activity and Specificity—A Structural and Biochemical Approach

**DOI:** 10.1371/journal.pone.0158024

**Published:** 2016-06-28

**Authors:** Sruti Dutta, Debi Choudhury, Sumana Roy, Jiban Kanti Dattagupta, Sampa Biswas

**Affiliations:** Crystallography and Molecular Biology Division, Saha Institute of Nuclear Physics, 1/AF Bidhannagar, Kolkata, 700 064, India; Stanford University, UNITED STATES

## Abstract

Papain-like proteases contain an N-terminal pro-peptide in their zymogen form that is important for correct folding and spatio-temporal regulation of the proteolytic activity of these proteases. Catalytic removal of the pro-peptide is required for the protease to become active. In this study, we have generated three different mutants of papain (I86F, I86L and I86A) by replacing the residue I86 in its pro-peptide region, which blocks the specificity determining S2-subsite of the catalytic cleft of the protease in its zymogen form with a view to investigate the effect of mutation on the catalytic activity of the protease. Steady-state enzyme kinetic analyses of the corresponding mutant proteases with specific peptide substrates show significant alteration of substrate specificity—I86F and I86L have 2.7 and 29.1 times higher *k*_cat_/*K*_m_ values compared to the wild-type against substrates having Phe and Leu at P2 position, respectively, while I86A shows lower catalytic activity against majority of the substrates tested. Far-UV CD scan and molecular mass analyses of the mature form of the mutant proteases reveal similar CD spectra and intact masses to that of the wild-type. Crystal structures of zymogens of I86F and I86L mutants suggest that subtle reorganization of active site residues, including water, upon binding of the pro-peptide may allow the enzyme to achieve discriminatory substrate selectivity and catalytic efficiency. However, accurate and reliable predictions on alteration of substrate specificity require atomic resolution structure of the catalytic domain after zymogen activation, which remains a challenging task. In this study we demonstrate that through single amino acid substitution in pro-peptide, it is possible to modify the substrate specificity of papain and hence the pro-peptide of a protease can also be a useful target for altering its catalytic activity/specificity.

## Introduction

**Fig 1:**
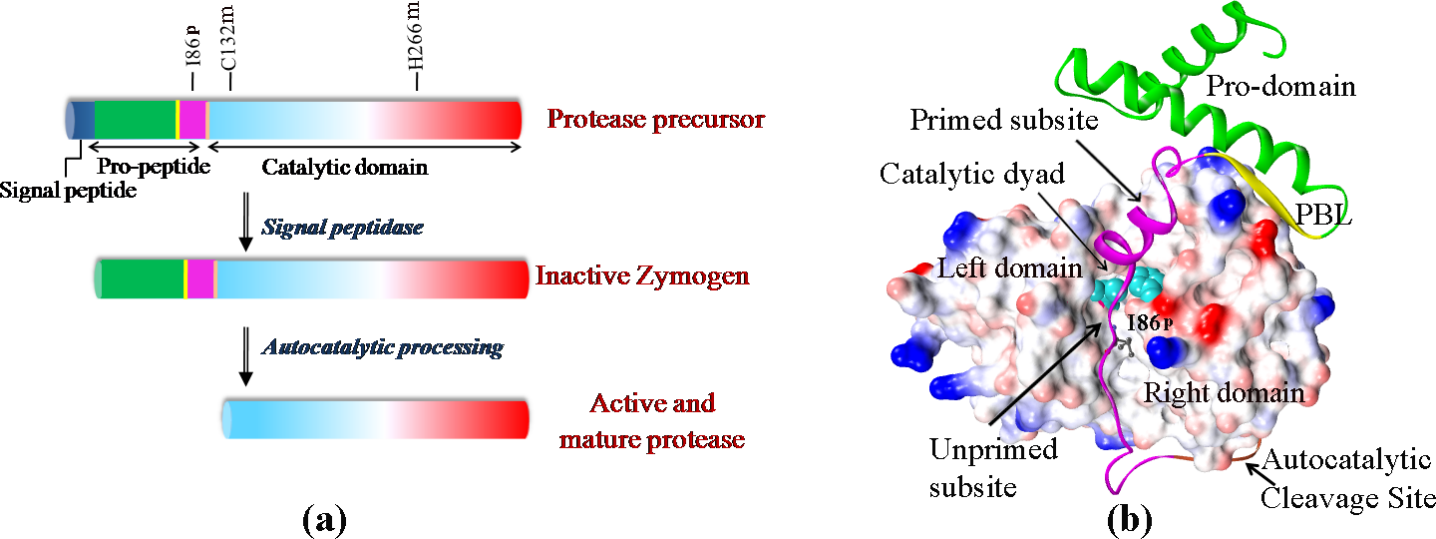
The structure of papain and its precursor form. **a**) A schematic representation of papain precursor and activation mechanism. **b)** Three dimentional structure of zymogen of papain (PDB ID: 3TNX); catalytic domain is represented as electrostatic potential surface with catalytic dyad residues represented as light-blue CPK model. The pro-peptide is represnted in ribbon. The color scheme used for (a) and (b) are: nevyblue, signal peptide; green, N-terminal pro-domain; yellow, PBL binding β-strand; magenta, the polypeptide blocking the substrate binding subsites; orange, autocatalytic cleavage site; blue-red gradient; catalytic domain. ‘m’ and ‘p’ tags in the sequence number represent mature and pro-peptide region respectively.

Generally a cysteine protease of papain family (clan C1A) is synthesized in the cell as a precursor protein, containing three parts; a signal peptide, a pro-peptide and a mature catalytic domain (Fig 1A). The signal peptide, which is required to transport the enzyme to endoplasmic reticulum, is cleaved off by signal peptidases after transportation [1]. The pro-peptide works as an intramolecular chaperone which catalyses the folding of the catalytic domain of the cognate protease [1, 2]. It also functions as an inhibitor of the protease by rendering the enzyme in a catalytically latent condition in its zymogen form [3]. Like most other proteases, the spacio-temporal regulation of proteolytic activity is maintained by N-terminal pro-peptide in a papain-like cysteine protease [1–3]. This N-terminal part of the pro-peptide forms a domain (pro-domain) consisting of three α-helices and a short β-strand and the rest of the pro-peptide is in extended conformation (‘extended pro-peptide’) (Fig 1B). This extended pro-peptide blocks the active site cleft by positioning itself in a non-productive orientation (reverse of a natural substrate) thereby preventing access of substrates to the catalytic site of the enzyme [4,5]. The ‘pro-domain’, which acts as a scaffold, is structurally and amino-acid sequence wise similar in all cathepsin-L type papain-like proteases [5–9]. In contrast, amino acid sequence and length of the ‘extended pro-peptide’ vary among the proteases in the family. Structural comparisons of the zymogens of the family [5–9] reveals that binding of pro-peptide to its cognate catalytic domain is governed by interactions in two key areas of catalytic domain: at the pro-peptide binding loop (PBL) of right domain of the mature protease part (or catalytic domain) (Fig 1B) and at the substrate binding subsites of the catalytic-domain (Fig 1B). The short β-strand of pro-domain docks into a pocket of right-domain and forms a β-sheet with a β-strand of PBL (Fig 1B). The third helix of pro-domain interacts with the primed subsites and a part of the extended pro-peptide runs through unprimed subsites of the catalytic cleft. Therefore the extended pro-peptide region, having a variable amino acid sequence, is the major interacting zone of the associated catalytic domain during protein folding and these residues show specificity for the cognate protease.

Papain-like proteases play an important role in wide range of industrial applications [10, 11] and they are medically relevant in humans and human pathogens [12–13]. The specificity of these proteases is determined by interactions of well-defined S2 subsite of the protease with the side-chain of second residue (P2) from the scissile peptide bond of the substrate. Natural variations in amino-acids forming the S2 subsite cleft occur in members of the family which contribute to the specificity of individual proteases. There are reports on mutagenesis studies of these residues to alter specificity of enzymes [14–16].

In zymogen form of the protease, conformation of the catalytic cleft has a shape, size and charge, complementary to that of the pro-peptide region which blocks the catalytic cleft for the stability of the structure [4]. Therefore the specificity of the protease is likely to be dependent on the composition of amino-acid residues (or amino-acid sequence) of the pro-peptide blocking the catalytic cleft in zymogen [2] and it may be possible to alter specificity or to engineer tailor-made specificity by mutating the residue(s) of this region of pro-peptide of a protease. To understand the role of pro-peptide in determining the specificity of the cognate protease and the consequence(s) of this mutation(s) on proteolytic activity of a protease, we mutated a vital amino acid of the pro-peptide which blocks the specificity pocket (S2) of papain [9], the archetypal protease in the family, in its zymogen structure. Enzyme kinetic analyses show that the mutant enzymes have altered specificity. Subsequent mass spectrometry and far-UV CD analyses of the mature catalytic domain do not show any significant differences in masses and secondary structures of the mutants and wild-type proteases. Crystallographic studies of the mutant pro-enzymes also demonstrate no gross conformational changes in the catalytic cleft. It is therefore expected that some fine tuning of side-chains and main-chains of the residues of the catalytic cleft would occur to adopt the mutation in the pro-peptide which may be responsible for altered proteolytic activity and specificity of papain. Decoding details of micro-changes of conformation at the catalytic cleft, is a challenging task requiring atomic resolution structure of catalytic domain after zymogen activation.

The alteration of catalytic activity and specificity of an enzyme is a central issue in protein biochemistry and biotechnology. Generally the trend is to alter activity and specificity by introducing mutations in the catalytic sites and substrate binding subsites. This study reports that pro-peptide of the proteases can also be an important target for protein engineering to alter activity and/or specificity.

## Materials and Methods

### Site-directed mutagenesis, expression and purification of mutants of papain

In our earlier studies, we reported a thermostable mutant of papain (K281R/V139S/G143S; pro-papain numbering) which showed proteolytic activity comparable to that of the wild-type papain [17]. The X-ray structure of the thermostable mutant [9] revealed that positions of these three mutations were not in proximity of either the catalytic cleft or the interface of pro-peptide/catalytic domain and it was also observed that the mutation did not induce any structural change(s) in the catalytic cleft. So, this papain mutant, with an advantage of higher stability, can be treated as a model system to understand the factors that regulate the catalytic activity, specificity and zymogen activation of the wild-type papain. This wild-type (thermostable) papain has been designated as WT (TS). In the present study this mutant cDNA of pro-papain, cloned in pET30 Ek/LIC vector [17], has been used for introducing mutations in its pro-peptide region.

Three mutants, I86F, I86L and I86A were generated using the standard protocol of the QuikChange^®^ Site-Directed Mutagenesis Kit (Agilent Technologies, USA) with specifically designed complementary primers containing the desired point mutations (Table 1). All plasmid DNA were sequenced and the correctness of the mutations was verified. Plasmids bearing the desired mutations were transformed into *E*. *coli* BL21 (DE3) expression strain for recombinant protein production. Expression and purification of mutant proteins were carried out as described earlier for wild-type [17, 18]. Briefly, proteins expressed as inclusion bodies, which were solubilized with urea, purified by Ni-NTA affinity chromatography under denaturing conditions and refolded by dilution method. The refolded purified protein was concentrated by Amicon Ultra-4 (10 kDa cut-off) for further studies.

**Table 1:** Oligonucleotides used for site-directed mutagenesis.

Mutants	Primers
I86F	F 5' GAAAAGTATACTGGTTCTTTTGCTGGAAATTATACAACAACC 3';R 5' GGTTGTTGTATAATTTCCAGCAAAAGAACCAGTATACTTTTC 3'
I86L	F 5' CAAAGAAAAGTATACTGGTTCTCTTGCTGGAAATTATACAACAACC 3';R 5' GGTTGTTGTATAATTTCCAGCAAGAGAACCAGTATACTTTTCTTTG 3'
I86A	F 5' CAAAGAAAAGTATACTGGTTCTGCTGCTGGAAATTATAC 3';R 5' GTATAATTTCCAGCAGCAGAACCAGTATACTTTTCTTTG 3'
C132A	F 5' CAGGGTTCTTGTGGTAGTGCGTGGGCATTCTCAGCTGTTG 3';R 5' CAACAGCTGAGAATGCCCACGCACTACCACAAGAACCCTG 3'
C132S	F 5' CAGGGTTCTTGTGGTAGTTCGTGGGCATTCTCAGCTGTT 3';R 5' AACAGCTGAGAATGCCCACGAACTACCACAAGAACCCTG 3'

Underlined sequences represent the designated mutation for the target amino acid residue.

### Zymogen activation

The purified refolded mutant pro-enzymes (5–10 μg) were activated in 100 mM Na-acetate buffer pH 5.0, 2 mM EDTA with cysteine (20 mM) as an activator using a protocol similar to that of the wild-type (thermostable) protein [18]. The temperature and time of activation for each mutant was optimized.

### Gelatin gel zymography and Azocasein assay

The recombinant refolded mutant proteases (I86F, I86L and I86A) and the wild-type (TS) protease were analyzed for protease activity by substrate gel zymography using 0.1% gelatin as a substrate using a protocol described previously [17].

The proteolytic activity of WT (TS) and mutant proteases was measured individually with substrate azocasein, for comparison. For this, the hydrolysis of azocasein by the activated protease was determined by increase in absorbance at 366 nm against time in 100 mM Na-acetate buffer, pH 6.5, containing 2 mM EDTA. One enzyme unit was defined as the amount of protease required to release 1 μg of soluble azopeptides/min (considering A^1%^_366nm_ = 40 for soluble azo-peptide). The specific activity was the number of units of activity per milligram of protein.

### Determination of steady-state enzyme kinetic parameters

Proteolytic activity of the mutants and WT (TS) was measured spectrophotometrically with different chromogenic peptidyl substrates containing p-nitroanilide (Sigma Aldrich, USA) (Table 2). The pro-enzymes were activated as described earlier and added to a reaction mixture (50 mM Na-accetate pH 6.5, 2 mM cysteine, 2 mM EDTA and 0.1% Brij 35) with the substrate to a volume of 0.5 ml for measuring activity. The initial velocity of the reaction was determined by continuously monitoring the release of p-nitroaniline (pNA) from the substrates by measuring absorbance at 410 nm using an extinction coefficient of 8800 M^-1^ cm^-1^ for pNA [19] on a UV/Vis spectrophotometer (Nicolet Evolution 100; Thermo Electron Corporation, Rockville, MD, USA). Kinetic parameters *K*_m_ and *V*_max_ values for each enzyme-substrate combination were obtained from nonlinear fitting of the Michaelis–Menten curve using the software Graphpad PRISM version 6 (http://www.graphpad.com/prism) with substrate concentration ranges spanning well below to well above the *K*_m_ (4–5 times of *K*_m_) unless precluded by substrate solubility problem. The *k*_cat_ value was determined by using the equation *k*_cat_ = *V*_max_ / [E]_T_ where [E]_T_ is the total concentration of the active enzyme, the values of which were measured by active-site titration with the irreversible inhibitor, E-64 which binds in a 1:1 molecular ratio at the active sites of most cysteine proteases [20]. Briefly, a fixed concentration of pro-enzyme was incubated in activation buffer (100 mM sodium acetate buffer pH 4.0 containing 2 mM EDTA and 20 mM cysteine) at optimised temperature and time in order to obtain mature mutant proteases. Immediately after conversion, the irreversible inhibitor, E-64 was added and the enzyme-inhibitor mixture incubated at RT for ~5 min. Subsequently a pre-determined amount of N-Benzoyl-Phe-Val-Arg- p-nitroanilide in 50 mM sodium acetate buffer pH 6.5 containing 2 mM EDTA, 2 mM cysteine and 0.1% Brij 35 was added and the release of pNA was monitored for 20 min at 410 nm. This process was repeated with increasing concentrations of E-64 until the residual activity reached zero. This residual activity of the enzyme was plotted against the inhibitor concentration. Total enzyme concentration [E]_T_ is equal to the molar concentration of E-64 required to inhibit the total proteolytic activity of the enzyme. The values of active enzyme concentrations were found to be in the range of 15–65 nM. Three independent kinetic determinations were made to calculate means and standard deviations for the reported *K*_*m*_ and *k*_cat_ values (Table 2 and S1 Text).

**Table 2:** Kinetic constants for peptidyl p-nitroanilides. Each kinetic constant with standard deviation is the average value of three independent experiments. Highest *k*_cat_/ *K*_m_ (M^-1^ sec^-1^) value for each substrate is shown in bold.

Enzyme	Substrate	*k*_cat_ (sec^-1^)	*K*_m_ (μM)	*k*_cat_/ *K*_m_ (M^-1^ sec^-1^)
WT (TS)	Pyroglutamyl-Phe-Leu-p-nitroanilide	2.91±0.05	261.57±4.07	11143.8±291.51
I86F	Do	2.80±0.14	94.81±6.79	**29546.5±754.85**
I86L	Do	2.33±0.08	940.50±87.50	2492.08±309.95
I86A	Do	1.22±0.01	344.20±7.05	3556.28±91.04
WT (TS)	N-benzoyl-Arg-p-nitroanilide	0.41±0.01	3738.67±10.02	**110.55±1.25**
I86F	Do	0.3±0.02	3443.5±9.19	85.68±6.39
I86L	Do	No detectable activity	No detectable activity	No detectable activity
I86A	Do	No detectable activity	No detectable activity	No detectable activity
WT (TS)	N-Benzoyl-Phe-Val-Arg- p-nitroanilide	4.07±0.04	57.93±1.77	70320.09±1915.10
I86F	Do	14.08 ±1.37	244.43±12.79	57520.18±3126.76
I86L	Do	6.21±0.19	86.48±6.12	**71976.41±3013.93**
I86A	Do	3.39±0.17	117.10±11.76	29050.58±1469.39
WT (TS)	D-Val-Leu-Lys- p-nitroanilide	0.67±0.04	545.93±21.42	1226.99±111.33
I86F	Do	No detectable activity	No detectable activity	No detectable activity
I86L	Do	3.96±0.11	110.63±4.66	**35800.66±579.85**
I86A	Do	No detectable activity	No detectable activity	No detectable activity
WT (TS)	N-Succinyl-Ala-Ala-Ala- p-nitroanilide	No detectable activity	No detectable activity	No detectable activity
I86F	Do	No detectable activity	No detectable activity	No detectable activity
I86L	Do	No detectable activity	No detectable activity	No detectable activity
I86A	Do	No detectable activity	No detectable activity	No detectable activity

### Analysis of mature protein by mass spectrometry

To generate mature proteases for mass spectrometry (MS), the refolded WT (TS) and mutant pro-enzymes (5–10 μg) were activated in 50–100 mM Na-acetate buffer pH 4.0 containing 2 mM EDTA using 5 mM DTT as an activator, for 25 min at 45°C. Immediately after incubation, the mature proteases were inactivated by 10 μM E-64 to stop further autocatalytic degradation. The completion of maturation process was checked by SDS-PAGE analyses. The protein samples after maturation were extensively exchanged into 50 mM ammonium bicarbonate, pH 7.8 and freeze-dried for intact mass analyses. The freeze-dried samples were re-suspended in 50 mM ammonium bicarbonate, pH 7.8 and an aliquot of these protein samples were mixed with 10 mg/ml sinapinic acid in 30% acetonitrile and 0.1% trifluoroacetic acid and subsequently analyzed by MALDI-TOF MS (Ultraflextreme, Bruker Daltronics) in linear mode to determine masses.

In another experiment, MALDI-TOF MS was used with peptide mass fingerprinting of the mature domain to understand the cleavage site(s) during autocatalytic activation of zymogens. The most intensely stained region at the center of the mature protein bands of WT (TS) and I86F mutant was cut and in-gel digestions were performed with porcine trypsin (Sigma-Aldrich, USA) using a protocol described by Shevchenko et al [21]. The cysteines were carbamido-methylated by iodoacetamide. Sample preparation for MALDI-TOF MS analyses from the extracted tryptic digested peptides was similar to that of the intact mass analyses. The instrument parameters were set as follows: detector, reflector mode; accelerating voltage, 25 kV; delay time, 1 μs; laser intensity, 2500. Acquisition was made in the range m/z 700–3500 Da. A total of 500 shots were performed per spectrum, and 20–25 spectra were accumulated per sample to increase the signal to noise ratio. Spectra were acquired in the positive ion mode. External calibration was performed using the mono isotopic masses of the singly charged ions produced by a solution containing Bradykinin fragment 1–7 (757.3997Da), Angiotensin I (1296.6848 Da), angiotensin II (1046.5423 Da), Substance P (1347.7354 Da), Bombesin (1619.8223 Da), ACTH fragment 1–17 (2093.08620 Da), ACTH fragment 18–39 (2465.1989 Da). Internal calibration was performed using trypsin autolysis of keratin peaks. Spectra were processed and analyzed using the program FlexAnalysis version 3.4 (BrukerDaltonics). In order to confirm the mature domain, MALDI-MS/MS analyses were performed. The resulting peptide map was searched against the NCBI database for matching proteins using the MASCOT search engine. MS/MS data processing was done by using LIFT method.

### Circular dichroism (CD) spectroscopy

The mature protease samples in 25 mM ammonium bicarbonate, pH 7.8, used for intact mass analyses, were also checked for secondary structural aspects by far-UV CD spectroscopy. A Jasco J-815 circular dichroism spectrophotometer (Jasco, Inc.) was used to measure far UV CD spectrum for inactivated mature proteases described in previous section. Measurements were conducted in triplicate with 1 nm steps in a quartz cuvette with 0.1 cm path length and spectra were recorded at a speed of 100 nm/5 min between 190–250 nm.

### Generation of catalytic mutant of I86F/L/A for structural studies and crystallization

Catalytic mutants of I86F, I86L and I86A were generated by replacing the active site cysteine to alanine (C132A) to produce inactive protease molecules so as to avoid autocatalysis for structural studies. For mutagenesis and expression of the mutants, same protocol has been used as described earlier. It was observed that although the expression level of all the proteins were similar as was the yield of the denatured protein after purification with Ni-NTA affinity chromatography under denaturing conditions, the I86A mutant was found to degrade/ breakdown during refolding. Altering refolding conditions did not help and so we decided to mutate the active site Cys of I86A to Ser (C132S) to see whether we could obtain a properly folded inactive mutant of I86A for structural studies.

Refolded I86F/L-C132A and I86A-C132S proteins were concentrated and purified further by gel-filtration chromatography on a Sephacryl S-100 (Sigma-Aldrich, USA) column that had been pre-equilibrated with 20 mM Tris–HCl pH 8.0, 200 mM NaCl and 1% glycerol. The protein was eluted in the same buffer and the peak fractions were collected and concentrated to 10–15 mg/ml in same column buffer. The purity of the proteins was checked by 15% SDS–PAGE analysis. The I86A-C132S mutant showed two bands with almost equal intensity in SDS-PAGE after refolding and subsequent gel filtration (data not shown). Spontaneous break down of this mutant could not be avoided. Therefore, crystallization trials were made for I86F-C132A and I86L-C132A mutants only.

Initial crystallization trials for I86F/L-C132A mutants were carried out by hanging drop vapour diffusion method using Structure Screen 1 and 2 (MD1-01 & MD1-02) of Molecular Dimensions Limited (United Kingdom). Diffraction quality crystals were obtained with 8% PEG 4000 in 100 mM Na-acetate (pH 4.6) (Condition 6 of MD1-01) at 20°C. The crystals were cryo-protected with the above precipitant containing 15–30% glycerol and flash-frozen in liquid nitrogen.

### Data Collection and structure determination

X-ray diffraction data-sets were collected from flashed frozen crystals at 100K on a Mar-CCD detector at BM14 beam-line ESRF, Grenoble, France. The diffraction data were processed and scaled using HKL2000 program [22]. Molecular replacement were carried out using PHASER [23] in PHENIX with the WT (TS) structure (PDB ID: 3TNX) as a model. For I86F and I86L mutants, the Ile86 has been replaced by an Ala residue in the model. The pro-peptide region which blocks the catalytic cleft were traced from |F_o_|-|F_c_| omit map for I86F and I86L mutant. Iterative rounds of model building with Coot [24] and refinement using PHENIX [25] and REFMAC5 [26] were performed using a translation-liberation-screw model of the atomic displacement parameters. Co-ordinates and structure factors were deposited in protein data bank (PDB) with accession codes 4QRV and 4QRG for I86F and I86L mutants respectively. Refinement and diffraction data statistics are summarized in Table 3.

**Table 3:** Data collection and refinement statistics.

	I86F	I86L
PDB code	4QRV	4QRG
**Data statistics**		
Beam-line	BM14, ESRF	BM14, ESRF
Wavelength (Å)	0.95	0.97
Resolution range (Å)	37.13–1.978 (2.049–1.978)	39.34–2.497 (2.586–2.497)
Space group	P 1 21 1	P 1 21 1
Unit cell (a, b, c in Å and β in°)	42.67 74.27 116.33 92.85	42.72 75.22 116.46 93.21
Total reflections	285449	99119
Unique reflections	50432 (4718)	25611 (2413)
Multiplicity	5.7 (5.4)	3.9 (3.4)
Completeness (%)	99.23 (93.09)	99.44 (94.63)
Mean I/σ(I)	16.25 (2.55)	11.23 (1.96)
Wilson B-factor (Å^2^)	27.87	33.85
R-merge	0.076 (0.58)	0.10 (0.68)
**Refinement statistics**		
R-work	0.1984 (0.2441)	0.1567 (0.2183)
R-free	0.2494 (0.2983)	0.2225 (0.2735)
Number of non-hydrogen atoms	5191	4915
Protein	4818	4799
Ligands	4	3
Water	369	113
Protein residues	603	602
RMS (bonds, Å)	0.008	0.008
RMS (angles,°)	1.01	1.08
Ramachandran favored (%)	97	95
Ramachandran allowed	2.8	4.3
Ramachandran outliers (%)	0.2	0.7
Average B-factor (Å^2^)	37.20	49.70
Protein	36.90	50.00
Ligands	64.20	73.70
Solvent	41.80	37.00

Statistics for the highest-resolution shell are shown in parentheses.

R-merge = SUM ((I—<I>) ^2^) / SUM (I ^2^)

R_work_ = Σ׀ Fo − Fc׀∕Σ Fo. R_free_ is the cross-validation R factor for the test set (5%) of reflections omitted in model refinement.

## Results and Discussion

### Expression, purification, zymogen activation and mature enzyme formation of the mutants and WT (TS) papain

**Fig 2:**
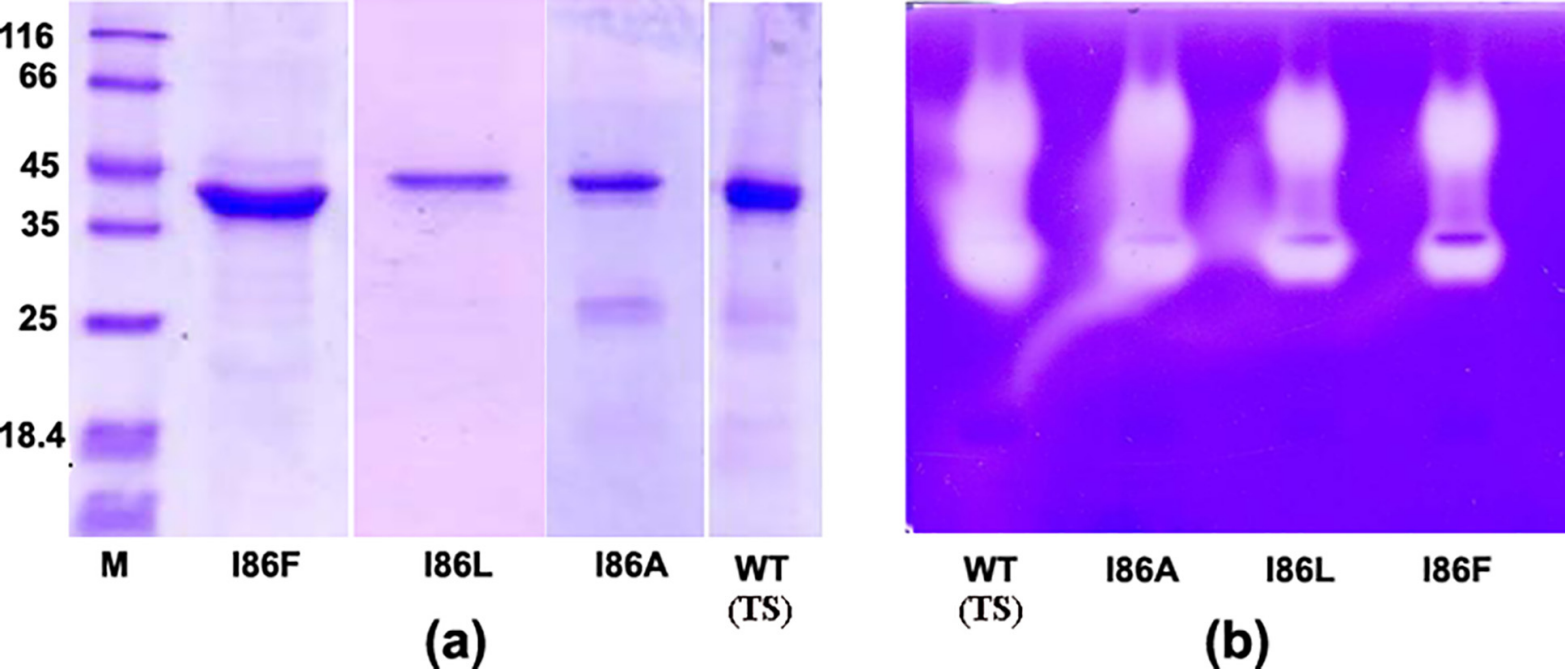
SDS-PAGE analyses of the proteases. **a)** Purified pro-proteases. **b)** Gelatin gel zymography for WT (TS) and three mutants. 2μg pro-proteases were loaded in each lane. The lanes of each gel are marked at the bottom. M lane is for molecular weight marker whose values in kDa are given at the left panel.

**Fig 3:**
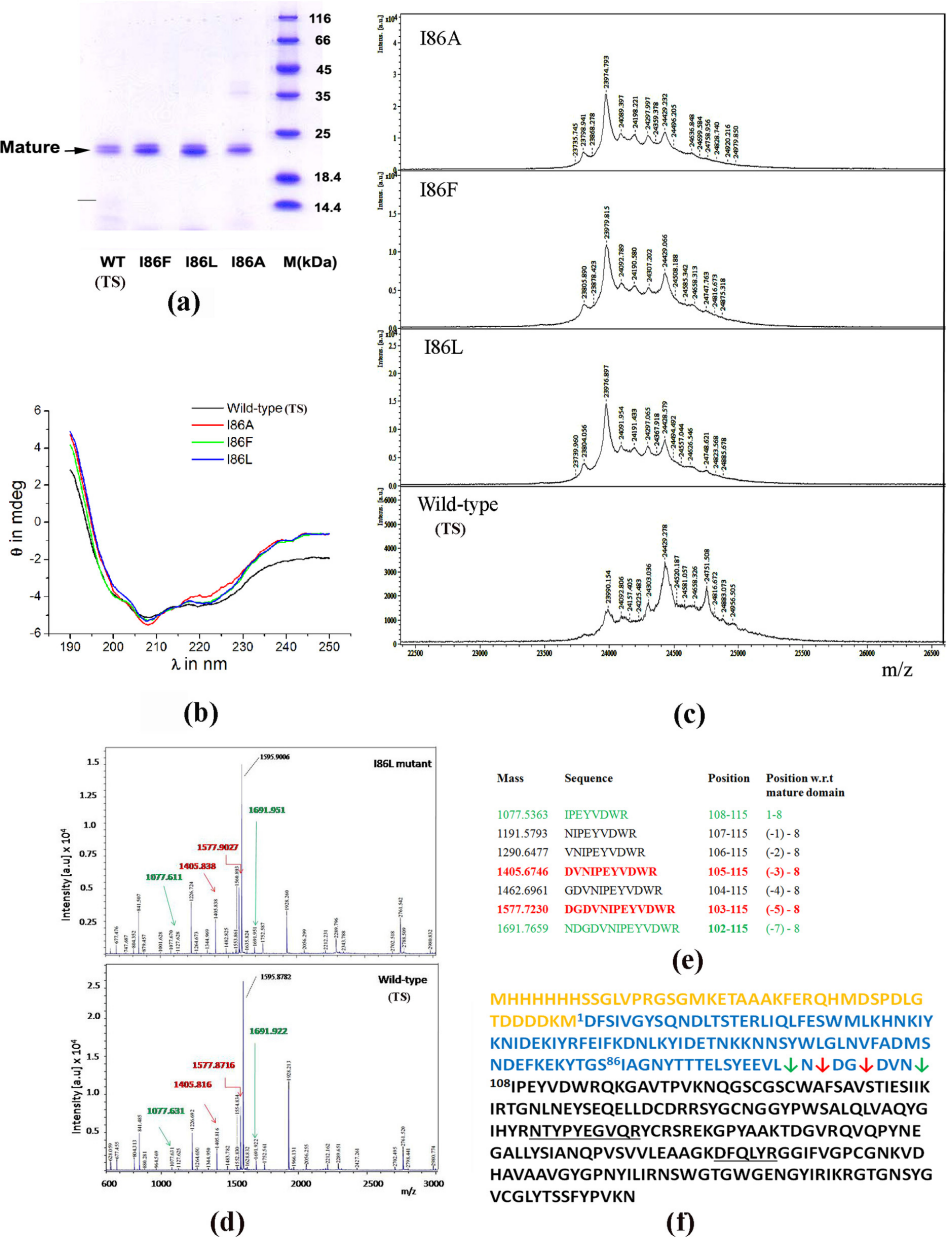
Analyses of mature proteases. **a)** Reducing SDS-PAGE analysis of the generated mature proteases. **b)** Far-UV CD spectra of mature proteases in the range 190 to 250 nm. **c)** MALDI-TOF MS spectra of mature proteases as shown in the SDS-PAGE (panel a). **d)** Peptide mass finger printing analyses after trypsin in-gel digestion of I86L and WT (TS) mature protease. **e)** Theoretical mass and sequence of the N-terminus of expected cleavages upto 7 residue downstream of the N-terminus of mature papain from plant latex. Sequences marked in red and green correspond to the peaks marked by same colored arrows in panel d. Red and green arrows represent high and low intensity peaks respectively in panel d. **f)** Sequence of the recombinant WT (TS); yellow, blue and black colour represent vector tag, pro-peptide and mature domain sequence respectively. Red and green arrows indicate cleavages corresponding to the peptide masses indicated in the same color in MS experiment (panels d and e). The sequences which matched with mature domain obtained from MS/MS analyses are underlined.

Expression, purification and refolding efficiency of three mutants, I86F, I86L and I86A, generated by site directed mutagenesis using specifically designed primers (Table 1), are similar to that of WT (TS) (Fig 2A and 2B). Zymogen activation conditions of the three mutants are almost similar to that of WT (TS) papain [18]. Mature proteases generated through auto-activation of cognate zymogens have molecular weights around 24 kDa for all three mutants and WT (TS), as observed from SDS-PAGE analyses (Fig 3A). This value is very close to the molecular weight of papain (UNIPROT ID: P00784) isolated from plant latex. We also observed that all the mature proteases bear similar secondary structures as revealed from far-UV CD spectra (Fig 3B).

It is reported that *in vitro* activation process does not always generate a distinctly single species of mature protein for papain-like proteases [27, 28]. In order to determine whether similar mature proteases are generated from pro-peptide mutants of papain and the WT (TS), we performed intact mass analyses of the mature proteases. MALDI mass spectrometry results demonstrate that mature proteases of the mutants I86F, I86L and I86A have main species around 23.97 kDa (this value of mass of the mature proteases include the mass of E-64, 0.357 kDa used for inhibition of mature proteases), which implies a longer mature domain compared to papain isolated from plant latex. In contrast, WT (TS) has a main peak at 24.42 kDa which also corresponds to a longer mature protease (Fig 3C). There are some minor peaks of mature proteases having their masses in the range 24.7–23.7 kDa, indicative of multiple cleavage sites. Since mature domains of three mutant proteases have almost similar mass spectra and they slightly differ from WT (TS), we performed in-gel tryptic digestion of one mutant, I86L and WT (TS) to compare exact masses of the mature domain of the two. The peptides from digestion of the excised band of WT (TS) and I86L mutant were analyzed by MALDI-TOF. The peptide masses in the map, shown in Fig 3D, were searched against the sequence of mature catalytic domain of papain (S1 Text). It is observed that there are 9 peptides in each protein which match peptide masses from catalytic domain obtained from theoretical trypsin digestion. Further, MS/MS analyses confirm the sequence of 2 peptides in the catalytic domain region (S1 Text and Fig 3F). The peptide mass spectra of WT (TS) and I86L confirm two peaks with higher intensity corresponding to cleavage sites 3 and 5 amino acids upstream of the correct N-terminus of *in vivo* processed mature papain from plant latex (Fig 3E and Fig 3F). There are also two minor peaks of masses corresponding to cleavages at the exact N-terminus and 6 amino acids upstream (Fig 3D, Fig 3E and Fig 3F) of it. Exact mass analyses do not show any significant change in mass spectra (Fig 3D) between WT (TS) and I86L mutant. This result implies that autocatalytic cleavage occurs at multiple sites over a range of polypeptide stretch of 1–6 amino acid length. It is well established that papain has a broad specificity for hydrophobic residues. The C-terminal portion of pro-peptide, containing number of hydrophobic residues, is less structured with high temperature factors [9] and accessible for proteolysis (Fig 1B). Hence cleavage may occur at multiple sites and generate number of mature proteases with closely related molecular weights. In the three dimensional structure of the zymogen of WT (TS) papain [9], the potential cleavage site is more than 30 Å away from the catalytic site (Fig 1B) and 21 residues downstream of the pro-peptide residue I86. It is therefore unlikely that enzyme kinetic parameters for *in vitro* processed mature recombinant WT (TS) and mutant papain would be affected by the presence of 1–6 additional N-terminal amino acid residues. This structure-based presumption was validated from our earlier experimental studies [18] and by Vernet *et*. *al*.[27]—in both the cases it was demonstrated that the catalytic activity of *in vitro* processed mature recombinant papain is similar to that of the mature papain purified from plant latex. Multiple cleavage sites, generated by *in vitro* auto activation, were also reported earlier for papain [27] and human cathepsin L [28], a papain-like lysozomal protease. It was also demonstrated by Wiederanders *et*. *al*., [2] that *in vivo* processing is assisted by different exo-peptidases/dipeptidyl peptidase which perform the final trimming process of the ragged N-terminus up to ‘X-Pro’ N-terminal sequence of papain-like proteases. The final trimming process of the flanking N-terminus is likely to impart additional stability to the mature enzyme.

### Effects of mutation at the P2 position of pro-peptide on the proteolytic activity of the protease

**Fig 4:**
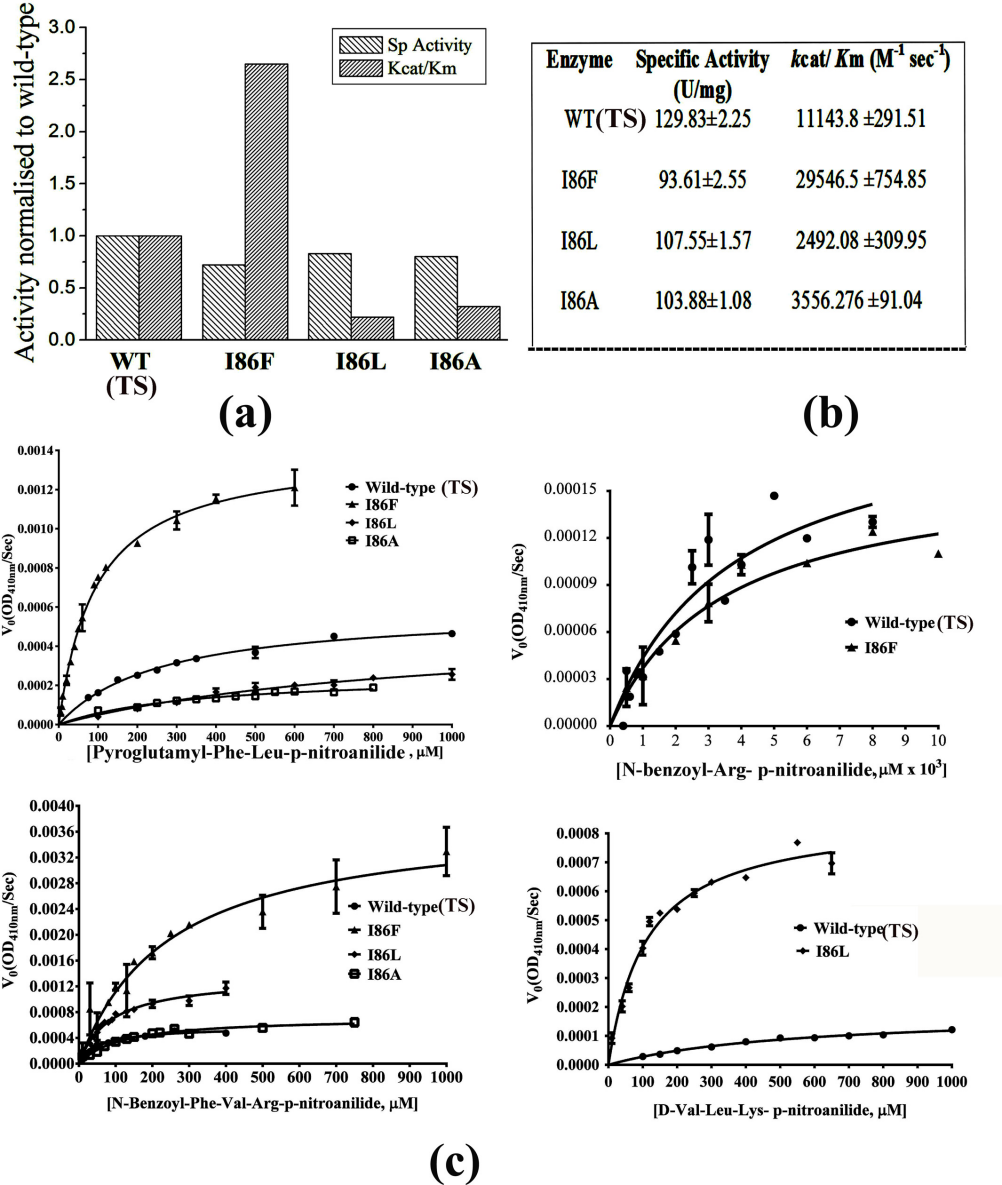
Effect of substitution at residue 86 of pro-peptide of papain on its proteolytic acivity. **a)** relative [normalised to WT (TS)] specific activity values against azo-casein and relative Kcat/Km values for the substrate Pyroglutamyl-Phe-Leu-p-nitroanilide. **b**) The actual values of the same are given in the table. **c)** Michaelis-Menten plot of proteolytic activity against different substrates for three mutants and WT (TS) papain. The error bar of each points represents standard error of the mean of replicates.

The gelatinolytic activities of the mutants have been checked by gelatin gel zymography. The mutant enzymes show slightly lower proteolytic activity compared to WT (TS) in the zymogram (Fig 2B). Notably, I86F shows lowest activity among mutant enzymes as observed in the zymogram. The caseinolytic activities of the proteases have been measured against azo-casein. I86F, I86L and I86A mutants show slightly reduced specific activity (71%, 83% and 80% respectively) against azo-casein compared to WT (TS) protease (Fig 4A & Fig 4B).

Though the proteolytic activities of the mutant enzymes do not vary much amongst themselves or in comparison with WT (TS) against protein substrates like casein or gelatin, their activities significantly differ against specific small oligo-peptide substrates (Fig 4A).

The steady state kinetic parameters *k*_cat_ and *K*_m_ values of three mutants and WT (TS) against five different oligo-peptide substrates indicate that mutants show differential specificity against different substrates, depending on the length and the type of amino acids at P1, P2 and P3 position of the substrate (Table 2); e.g. I86L mutant shows significant activity against D-Val-Leu-Lys- p-nitroanilide, having a leucine residue at P2 position of the substrate, which is 29.1 times higher than WT (TS) (Table 2). In contrast other two mutants (I86F and I86A) do not show any activity against the same substrate. A prominent difference in catalytic activity is observed for the substrate Pyroglutamyl-Phe-Leu-p-nitroanilide; mutant I86F shows 2.7 times higher activity than WT. For the same substrate, I86L and I86A show 0.2–0.3 times lower activity compared to WT (Table 2). The kinetic parameters further demonstrate that wild-type is active against almost all the substrates indicating its broad specificity. An isoleucine residue at 86^th^ position of pro-peptide of WT (TS) helps in casting a catalytic cleft which is likely to be responsible for its broad specificity. Mutations in this position by another hydrophobic residue, narrow down the specificity of the mutant proteases depending on the type of substituted amino acid (branched like leucine or aromatic like phenyl alanine). The kinetic data (Table 2) reveals that while I86F mutant prefers aromatic amino acid at the P2 position of the substrate, I86L prefers branched hydrophobic residues at the same position of the substrate.

### Overview of the zymogen structures of I86F and I86L mutants and comparison with the wild-type (thermostable) papain

**Fig 5:**
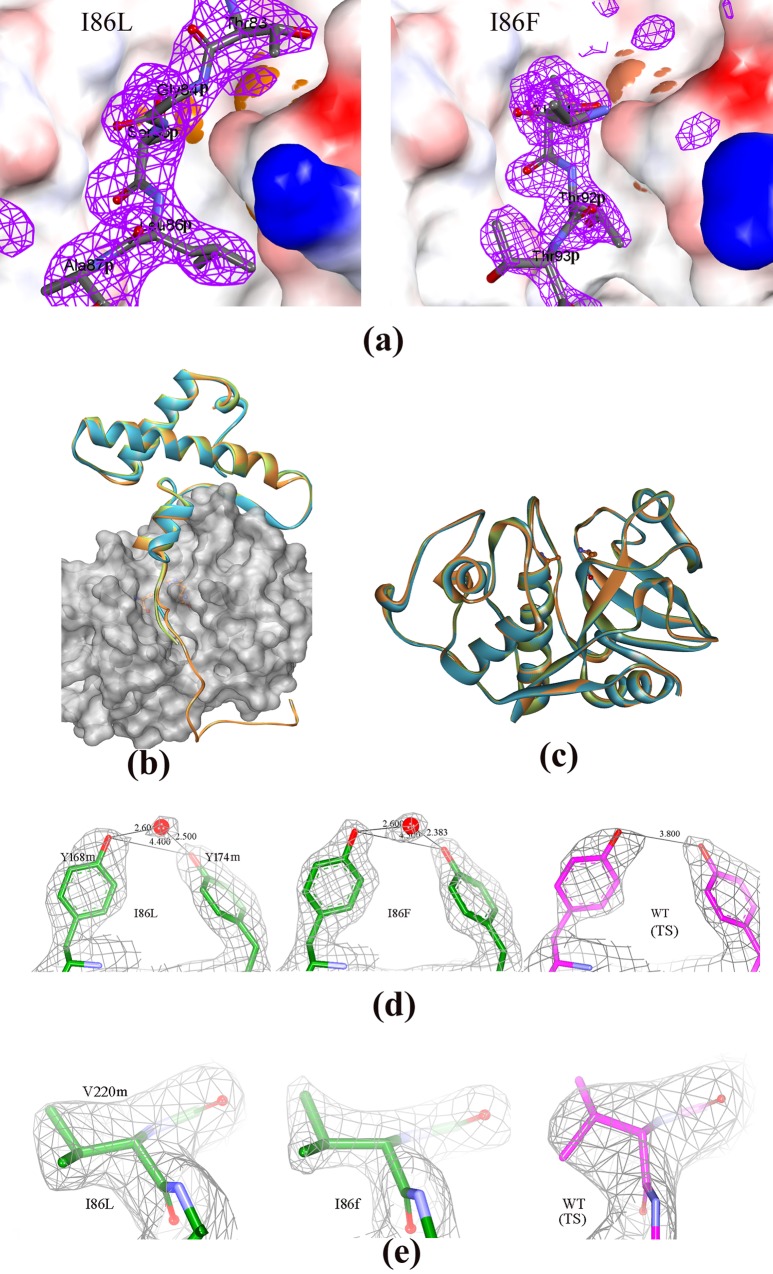
X-ray structures of the mutants and WT (TS). **(a)** Omit map (Fo-Fc) of I86L and I86F. The omit maps, contoured at 3.5σ level, were calculated by omitting the pro-peptide blocking the catalytic cleft. Catalytic domains of the two mutants are represented n electrostatic surfaces with the catalytic dyad in orange sphere superimposed therein. **(b)** Superposition of pro-peptide of I86L, I86F mutants and WT (TS). The propeptides are represented in ribbon. I86L, coloured in green; I86F, coloured in light blue; WT (TS) in light orange. The mature domain of the WT (TS) is in surface presentation with catalytic dyad in ball and stick. **(c)** Superposition of the mature catalytic domain with same color code used in (b). (**d)** and **(e)** The 2mFo-DFc electron density map at 1.5σ level of residues Tyr168, Tyr174 and Val220 in three proteins. The co-ordinates and structure factors of the WT (TS) have been taken from pdb_id 3TNX. ‘m’ and ‘p’ tags in the sequence number represent mature and pro-peptide region respectively.

**Fig 6:**
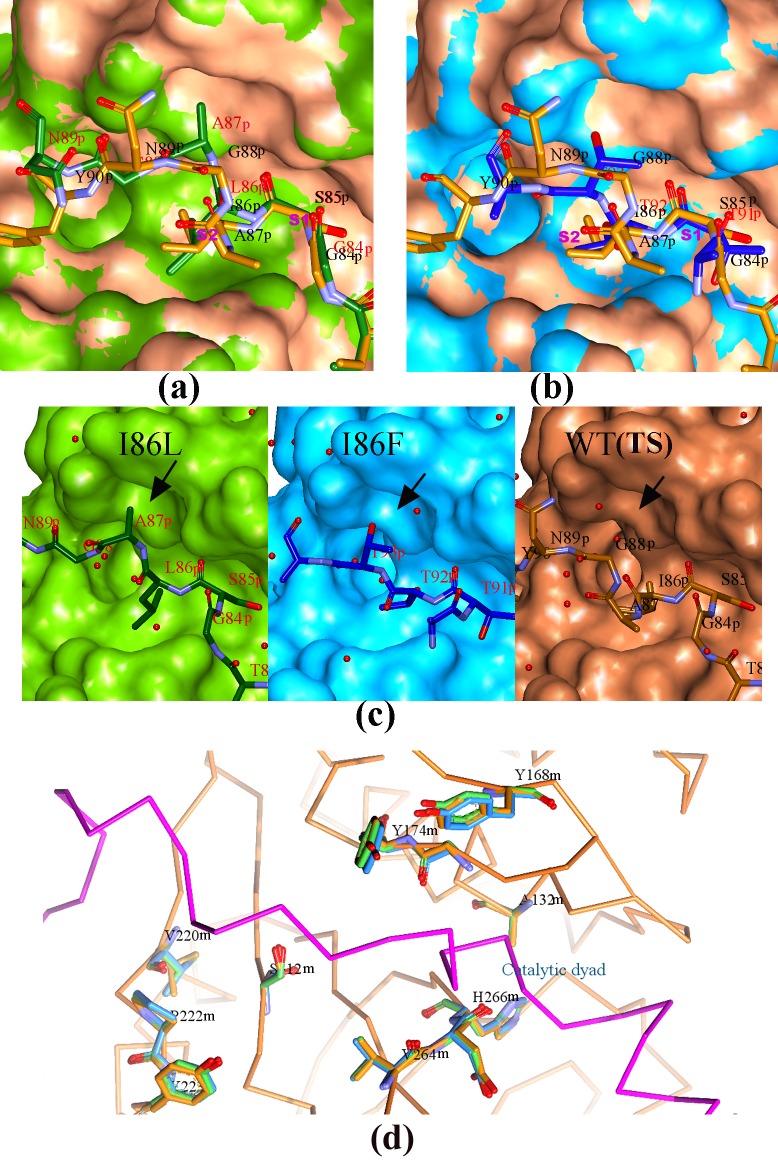
Structural comparison of catalytic cleft structures of I86F and I86L with that of the WT (TS). The catalytic domains are in surface presentation; I86L, coloured in green; I86F, coloured in light blue; WT (TS) in light orange. The pro-peptide parts blocking the catalytic clefts are presented as stick model with Carbon atoms coloured as their respective catalytic domain surface colour. **a)** and **b)** Superposition of I86L and I86F mutants with the WT (TS) respectively. **c)** Individual I86L, I86F and WT (TS) in a similar orientation of a) and b). The arrow indicates a cavity formed by two residues Tyr168 and Tyr174. **d)** Superposition of I86L, I86F and WT (TS) showing some residues of the catalytic cleft responsible for micro-changes in the cleft conformation of the mutants. The catalytic dyad residues are C132A and H266. The Cɑ trace is drawn on WT (TS)structure with mature domain in light orange and pro-peptide in magenta. The position of pro-peptide also represents the flow of substrate during Michaelis comlex formation.The co-ordinates and structure factors of the WT (TS) have been taken from pdb_id 3TNX. ‘m’ and ‘p’ tags in the sequence number represent mature and pro-peptide region respectively.

To understand the structural consequence of replacement of I86 residue in the pro-peptide of WT (TS) papain (PDB ID: 3TNX), we have determined the crystal structure of two mutants I86F and I86L. The mutants I86F and I86L crystallized in the same space group with similar unit-cell parameters as that of WT (TS) papain (Table 3). The zymogen structures of I86F and I86L mutants have been solved at 1.98 Å and 2.5 Å resolutions by molecular replacement methods using WT (TS) structure as a model. The refinement parameters of I86F and I86L mutants are given in Table 3. In both the structures, pro-peptide stretches blocking the respective catalytic clefts of the mature domains have clearly been observed in omit Fo-Fc electron density map (Fig 5A). Zymogen structure of I86L mutant shows that the mutated pro-peptide interacts with the mature catalytic domain almost in a similar manner like WT (TS) up to P2 position. In I86L mutant, the pro-peptide back-bone beyond P2, becomes totally extended (Fig 5B) compared to WT (TS) and this re-arrangement results Ala87 side-chain to orient differently in the mutant (Fig 5A & Fig 6C). Unlike WT (TS), no electron density is observed beyond P5 (Asn89) for the mutant. In I86F mutant, a different stretch of the extended part of pro-peptide occupies the catalytic cleft as compared to WT (TS) (90–94 compared to 84–89) (Fig 5A and Fig 6C). The poly-peptide stretch which blocks the catalytic cleft in WT (TS) (84–89 region) are seen to be disordered in this mutant. In the mature catalytic domain of the two mutants there are hardly any differences in the back-bone structure when compared with WT (TS) (Fig 5C). In the catalytic cleft, two minor but distinct differences in side-chain orientation with respect to the WT (TS) have been observed in Tyr168-Tyr174 pair and in Val220 (Fig 5D and Fig 5E). We initially pursued structure determination of mature domain of pro-peptide mutants after auto-catalytic activation to understand if these minor conformational changes are stable when pro-peptide is removed from the mature domain but we were unable to obtain suitable crystals for any of the samples. As it is described in the previous section, a mixture of mature enzymes with slightly different molecular weight is generated by auto-activation process and therefore homogeneous protein for crystallization could not be achieved which may be responsible for unsuccessful crystallization in spite of our best efforts. However it is already established [1–3] that unlike other proteases, the active conformation of the mature enzyme is pre-formed in the zymogen condition for papain-like proteases. For example, in WT (TS) papain, the rmsd of C-alpha atoms of the mature domain of zymogen structure (PDB ID: 3TNX) and native mature protease (purified from the plant latex; PDB ID: 9PAP) is of the order of 0.4 Å which implies that there is almost no conformational change of mature domain upon activation of the zymogen and this is true for other papain-like cysteine proteases for which structures of both the forms (zymogen and mature) are available [1–3]. Considering this fact we can presume that observed conformational changes at the catalytic site of the zymogen structures of the pro-peptide mutants I86L and I86F would be retained in their mature forms.

### Structural basis of altered specificities of I86F and I86L mutants

Enzyme catalysis is a dynamic process which includes binding of substrate(s), catalysis and release of product(s). Therefore it is difficult to envisage the structural factors responsible for substrate binding and catalysis from a static crystal structure. However X-ray structure can help in getting an overall idea about the process.

The structure of I86L shows that its S2 pocket is not suitable for accommodating side-chain of an aromatic residue due to slight rearrangement of the residues lining the pocket around Leu86. However side-chain of a branched hydrophobic residue can have a better fitting with strong hydrophobic interaction. The next residue, Ala87 points to a groove formed by Tyr174 and Tyr168 (Fig 6C and Fig 6D) and makes it deeper compared to WT (TS). A deeper pocket formed by the two aromatic residues Tyr174 and Tyr168 becomes a good binding region for a hydrophobic residue at P3 position of the substrate. This probably explains why I86L shows highest activity against the substrates **Val(P3)**-**Leu(P2)**-Lys(P1)-p-nitroanilide(P1’) and N-Benzoyl(P4)-**Phe(P3)**-**Val(P2)**-Arg(P1)-p-nitroanilide(P1’) having branched hydrophobic residue at P2 and hydrophobic residue at P3 position of the substrate.

In the structure of I86F mutant, it is revealed from the omit map (Fig 5A) around the catalytic cleft that three consecutive threonine residues (91, 92 and 93) occupy P1, P2 and P3 positions of pro-peptide (Fig 6C). The bulkier residue Thr91 at P1 position in the mutant compared to Ser85 of the WT (TS) creates a wider S1 subsite. In contrast, the S2 pocket in this mutant is not much altered (Fig 6B). Therefore I86F has a preference for aromatic side-chain at the P2 position of the substrate like WT (TS). However it can better accommodate a hydrophobic branched side chain like Leucine at the P1 position due to its wider S1 subsite. Papain usually prefers long polar side-chain (Arg/Lys/Gln) at P1 position [29] which can move outwards towards the solvent and does not require a deep S1 pocket while a hydrophobic residue is not a suitable candidate since it needs to be packed or buried in a hydrophobic S1 pocket. Micro-changes in the catalytic cleft conformation of I86F mutant set its specificity for a branched hydrophobic residue at P1 position and an aromatic ring at P2 of the substrate. Therefore the highest specificity of this mutant for the substrate Pyroglutamyl(P3)-**Phe(P2)**-**Leu(P1)**-p-nitroanilide(P1’) is most likely a combined effect of rearrangements in the S1 and S2 subsites.

In both the structures of I86L and I86F mutants, we have noticed two distinct changes in side-chain orientation of residues in the catalytic cleft which can be emphasized. The first one is a groove formed by residues Y168 and Y174. In the mutant structures, one water molecule bridges the hydroxyl atoms of the side-chains of the two Tyr residues which is absent in WT (TS) (Fig 5D and Fig 6D). It is not unusual that this bound water may influence substrate binding in the mutant proteins since water molecules play an important role in protein-protein interactions. The second alteration, which can categorically be highlighted, is the reorientation of V220 side-chain (Fig 5E and Fig 6D). This change also creates some alteration in the substrate binding region.

We were unable to crystallize I86A, since its catalytic mutant (I86A with C132A/S) was not stable enough for crystallization. From our experience of the structures of two mutants I86F and I86L, we can say that some micro-changes in the catalytic cleft might also be occurred here which is responsible for its altered substrate specificity. However the structural changes in the catalytic cleft is not straight forward to envisage, since the changes are dependent on the positioning of pro-peptide in the catalytic cleft in its zymogen form.

### Mechanistic implication for generation of template-directed conformational diversities leading to altered specificity of an enzyme

Most attempts to redesign enzyme activity are through mutating active site residues [15, 16]. Sometimes mutations are designed in the allosteric sites to alter allosteric regulation of enzymes and hence the enzymatic activity [30]. It is also reported by Shinde et. al. [31], that a point mutation in the pro-peptide of subtilisin altered the enzymatic activity of the protease. Shinde et. al. have proposed that the pro-peptide of subtilisin functions as an intra-molecular chaperone that imparts structural information during folding which is imprinted even when the pro-peptide is detached from the mature domain after zymogen activation [31]. However due to the absence of a three dimensional structure of the mutant subtilisin, exact mechanism of the imprinting process could not be visualized in their study. In the present study, we have shown that mutation(s) in the pro-peptide of papain generates active mature protease(s) with distinctly different substrate specificity (Table 2). The three dimensional structure of the zymogen of the mutant(s) further demonstrates that subtle conformational diversities in the catalytic cleft are achieved by slight adjustment of side-chain orientation and main-chain atoms of the residues forming the catalytic centre and of some residues lining the substrate binding subsites (Fig 6E). Considering the limitation of resolution of the present X-ray diffraction study and in absence of high resolution X-ray structure of mature catalytic protease after zymogen activation, the question arises: are these very small conformational changes of the catalytic cleft responsible for the alteration of substrate specificity in mutant enzymes or are there some other factors like alteration of activation cleavage sites which develop during or after activation process of the mutant enzymes? We have tried to address this problem indirectly by eliminating the second possibility. We performed intact mass analyses (Fig 3C) of the mature enzymes generated from the mutated and WT (TS) pro-enzymes and peptide mass finger printing of tryptic digested peptides (Fig 3D) which eliminate the possibility that the differences in substrate specificity among the mutants originate from the covalent differences such as a change in activation cleavage site. CD experiments (Fig 3B) further demonstrate that there are no gross changes of overall folding pattern of the mature mutant enzymes in comparison to WT (TS). It is well established [1–3] that in papain-like cysteine proteases, catalytic cleft fully acquires its conformation responsible for its functional activity in the zymogen form itself. Therefore based on the X-ray structures of the zymogen, thorough enzyme kinetic analyses (as discussed in materials and method) coupled with intact, tryptic digested peptide mass analyses and CD spectroscopy data of the mature enzymes one can hypothesize that mutation(s) at certain position(s) of pro-peptide may generate subtly different mature enzyme conformation with micro changes in substrate binding subsites leading to altered activity and specificity. At the same time we cannot conclusively say whether these minimal changes in conformations of identical sequence and similar overall fold of the catalytic domain are actually distinct and stable over time leading to distinct functional diversity, since we were unable to trap the active catalytic domain conformation after activation of the zymogens. Although the insights offered by the crystal structures of the zymogens do not wholly explain the basis for the distinct substrate specificity among the prodomain mutants, the kinetics data, specially for three substrates (Pyroglutamyl-Phe-Leu-p-nitroanilide, N-Benzoyl-Phe-Val-Arg- p-nitroanilide, D-Val-Leu-Lys- p-nitroanilide), clearly demonstrate that the differences are real and reproducible.

Conformational diversities within the identical amino-acid sequence of macro-molecules, related to functional diversities, are not unknown [32]. This conformational diversity is mainly achieved either by change of environment or by altered function of chaperone during folding [32]. A protein, where conformational diversity arises mainly from the side-chain re-orientation, has an energy minima similar but discrete as that of its native conformer in the energy landscape of co-ordinate space with small energy barrier in between [32] as it is likely to be happened in the pro-peptide mutants. At the same time it is also possible that the conformational diversity of the mutant proteases, leading to altered specificity, is transient and able to cross the energy barrier and may be inter-convertible over the time since we measured the activity immediately after auto-activation of the proteases. We do not ignore the possibility that these differences may have erased with time because technically it was not possible to store the proteases after activation for measuring the activity after certain time interval due to auto-proteolytic degradation of the active mature enzyme.

The results of the present study demonstrate the importance of pro-peptide in determining catalytic activity and specificity. Alteration of activity and specificity can be achieved by mutating residue(s) in the pro-region and these differences of activity/specificity are not caused by major fold differences. Due to resolution limit, we however could not unambiguously locate finer changes in the position of the back-bone atoms and atoms lining the oxyanion hole of the catalytic cleft which are important for the catalytic property of a protease. Findings of the present study have implications in enzyme re-design which suggest that the residues of pro-peptide could be another plausible target for generating tailor-made activity and specificity of a protease.

## Supporting Information

S1 Text Full description of the mass spectrometry data and details of calculation of enzyme kinetics parameters. (DOC)

## Abbreviations

E-64: 1-[L-N-(transepoxysuccinyl) leucyl]amino-4-guanidinobutane
EDTA: ethylene-diamine-tetra-acetic acid
DTT: Dithiothreitol
PBL: Pro-peptide binding loop
WT (TS): a thermostable variant of wild-type papain
