# Supplementary material for: Mutation in the Pro-Peptide Region of a Cysteine Protease Leads to Altered Activity and Specificity—A Structural and Biochemical Approach
Source: PLoS One. 2016 Jun 28;11(6):e0158024. doi: 10.1371/journal.pone.0158024 (PMC4924875; doi:10.1371/journal.pone.0158024)
Supplement: S1 Text — (DOC) [file pone.0158024.s001.doc]

**Analyses of mass spectrometry data**

**Sequence of recombinant wild-type:**

MHHHHHHSSGLVPRGSGMKETAAAKFERQHMDSPDLGTDDDDKM**^1^**DFSIVGYSQNDLTSTERLIQLFESWMLKHNKIYKNIDEKIYRFEIFKDNLKYIDETNKKNNSYWLGLNVFADMSNDEFKEKYTGS**^86^I**AGNYTTTELSYEEVLNDGDVN**^108^IPEYVDWR↓QK↓GAVTPVK↓NQGSCGSCWAFSAVSTIESIIK↓IR↓TGNLNEYSEQELLDCDR↓R↓SYGCNGGYPWSALQLVAQYGIHYR↓NTYPYEGVQR↓YCR↓SREK↓GPYAAKTDGVR↓QVQPYNEGALLYSIANQPVSVVLEAAGK↓DFQLYR↓GGIFVGPCGNK↓VDHAVAAVGYGPNYILIR↓NSWGTGWGENGYIR↓IK↓R↓GTGNSYGVCGLYTSSFYPVK↓N**

*Highlighted part is vector tag sequence; Red color represents pro-peptide part and black is for mature domain.

**↓** denotes the position of trypsin cuts in mature domain

**Table S1**: Expected peptides generated from trypsin digestion of mature domain. The peptide masses which matched with experimental data from MS analyses are shown in green.

| Theoretical mass | Position | [Artificial modification(s)](http://web.expasy.org/findmod/findmod_masses.html#Others) | Experimantal mass WT/I86L | Peptide sequence |
| --- | --- | --- | --- | --- |
| 2958.5570 | 219-245 |  |  | QVQPYNEGALLYSIANQPVSVVLEAAGK |
| 2760.2987 | 167-190 | Cys_CAM: 170 | 2761.520/2761.542 | SYGCNGGYPWSALQLVAQYGIHYR |
| 2702.1115 | 125-146 | Cys_CAM: 129, Cys-E-64 132 | 2702.495/2702.518 | NQGSCGSCWAFSAVSTIESIIK |
| 2156.9957 | 299-320 | Cys_CAM: 332 |  | GTGNSYGVCGLYTSSFYPVK |
| 2055.8924 | 151-167 | Cys_CAM: 163 | 2056.255/2056.299 | TGNLNEYSEQELLDCDR |
| 1928.0388 | 264-281 |  | 1928.213/1928.260 | VDHAVAAVGYGPNYILIR |
| 1596.7189 | 282-295 |  | 1595.9006/1595.8782 | NSWGTGWGENGYIR |
| 1226.5800 | 191-200 |  | 1226.692/1226.724 | NTYPYEGVQR |
| 1077.5363 | 108-115 |  | 1077.631/1077.670 | IPEYVDWR |
| 1105.5458 | 253-263 | Cys_CAM: 160 |  | GGIFVGPCGNK |
| 841.4203 | 247-252 |  | 841.485/841.507 | DFQLYR |
| 671.4086 | 118-124 |  |  | GAVTPVK |
| 606.3246 | 208-213 |  | 606.235/606.148 | GPYAAK |
| 547.2834 | 214-218 |  |  | TDGVR |

CAM (carbamido methylation)

E-64(active site cysteine inhibitor)

**Mass spectra of the tryptic digested peptides**:


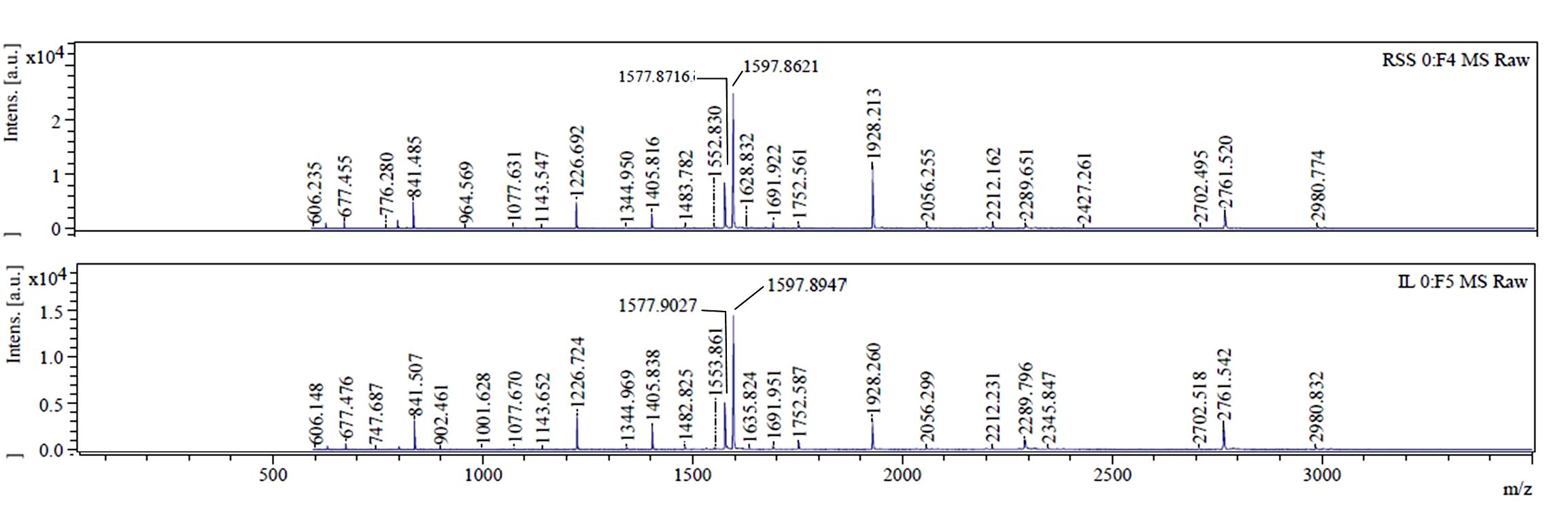


**MS/MS analyses:**

**Details of enzyme kinetic data**

**Wild-type with substrate L-pyroglutamyl-Phe-Leu-p-nitroanilide**

**Total concentration of the active enzyme, E_T_ = 23nM**

Data sets:

| [Substrate in µM] | Enzyme Activity (V_0_ in OD_410nm_/Sec), | | |
| --- | --- | --- | --- |
| 75. | 0.000128557 | 0.000145483 | 0.000139393 |
| 100. | 0.000169941 | 0.000154997 | 0.00016386 |
| 150. | 0.000224527 | 0.000224562 | 0.000235975 |
| 200. | 0.000258001 | 0.000253375 | 0.000243413 |
| 250. | 0.000280247 | 0.00028746 | 0.000267235 |
| 300. | 0.000296141 | 0.000328687 | 0.000322445 |
| 350. | 0.000324919 | 0.000353595 | 0.000331159 |
| 500. | 0.000381686 | 0.000387629 | 0.0003349 |
| 700. | 0.000443625 | 0.000457869 | 0.000451281 |
| 1000. | 0.000463142 | 0.000462728 | 0.000467708 |

| Michaelis-Menten |  |  |  |
| --- | --- | --- | --- |
| Best-fit values |  |  |  |
| Vmax | 0.0005893 | 0.0005994 | 0.0005808 |
| Km | 266.0 | 258.0 | 260.7 |
| Std. Error |  |  |  |
| Vmax | 1.762e-005 | 1.931e-005 | 3.694e-005 |
| Km | 19.27 | 20.38 | 40.50 |
| 95% Confidence Intervals |  |  |  |
| Vmax | 0.0005487 to 0.0006300 | 0.0005548 to 0.0006439 | 0.0004956 to 0.0006660 |
| Km | 221.5 to 310.4 | 211.0 to 305.0 | 167.3 to 354.1 |
| Goodness of Fit |  |  |  |
| Degrees of Freedom | 8 | 8 | 8 |
| R square | 0.9914 | 0.9899 | 0.9604 |
| Absolute Sum of Squares | 9.300e-010 | 1.168e-009 | 4.211e-009 |
| Sy.x | 1.078e-005 | 1.208e-005 | 2.294e-005 |
| Constraints |  |  |  |
| Km | Km > 0.0 | Km > 0.0 | Km > 0.0 |
|  |  |  |  |
| Number of points |  |  |  |
| Analyzed | 10 | 10 | 10 |

**I86F with substrate L-pyroglutamyl-Phe-Leu-p-nitroanilide**

**E_T_ = 57nM**

Data sets:

| [Substrate in µM] | Enzyme Activity (V_0_ in OD_410nm_/Sec) | | |
| --- | --- | --- | --- |
| 5. | 8.600000e-005 | 6.450000e-005 | 4.585060e-005 |
| 8. | 9.300000e-005 |  |  |
| 10. | 1.340000e-004 | 1.570000e-004 | 1.449420e-004 |
| 20. | 2.310000e-004 | 2.000000e-004 | 2.460180e-004 |
| 30. | 3.210000e-004 |  |  |
| 40. | 3.900000e-004 | 4.100000e-004 | 3.944620e-004 |
| 50. | 4.900000e-004 |  |  |
| 60. | 5.833900e-004 | 5.860000e-004 | 4.662490e-004 |
| 90. |  | 7.140000e-004 |  |
| 100. | 7.637900e-004 | 7.460000e-004 | 7.480000e-004 |
| 120. | 8.082530e-004 | 7.970000e-004 | 8.047450e-004 |
| 200. | 9.263420e-004 | 9.194140e-004 | 9.314670e-004 |
| 300. | 1.040000e-003 | 1.090000e-003 | 9.979790e-004 |
| 400. | 1.170000e-003 | 1.160000e-003 | 1.130000e-003 |
| 600. | 1.190000e-003 | 1.310000e-003 | 1.130000e-003 |

| Michaelis-Menten |  |  |  |
| --- | --- | --- | --- |
| Best-fit values |  |  |  |
| Vmax | 0.001392 | 0.001481 | 0.001338 |
| Km | 91.63 | 102.6 | 90.19 |
| Std. Error |  |  |  |
| Vmax | 2.794e-005 | 3.873e-005 | 4.189e-005 |
| Km | 5.094 | 7.444 | 8.540 |
| 95% Confidence Intervals |  |  |  |
| Vmax | 0.001331 to 0.001452 | 0.001395 to 0.001567 | 0.001243 to 0.001433 |
| Km | 80.53 to 102.7 | 86.02 to 119.2 | 70.87 to 109.5 |
| Goodness of Fit |  |  |  |
| Degrees of Freedom | 12 | 10 | 9 |
| R square | 0.9959 | 0.9941 | 0.9918 |
| Absolute Sum of Squares | 8.506e-009 | 1.081e-008 | 1.291e-008 |
| Sy.x | 2.662e-005 | 3.288e-005 | 3.788e-005 |
| Constraints |  |  |  |
| Km | Km > 0.0 | Km > 0.0 | Km > 0.0 |
|  |  |  |  |
| Number of points |  |  |  |
| Analyzed | 14 | 12 | 11 |

**I86Lwith substrate L-pyroglutamyl-Phe-Leu-p-nitroanilide**

**E_T_**=24.7 nM

| [Substrate in µM] | Enzyme Activity (V_0_ in OD_410nm_/Sec) | | |
| --- | --- | --- | --- |
| 100. | 4.260000e-005 | 5.000000e-005 | 2.551000e-005 |
| 200. | 7.400000e-005 | 9.470000e-005 | 7.304540e-005 |
| 300. | 1.040590e-004 | 1.203230e-004 | 1.130610e-004 |
| 400. | 1.560000e-004 | 1.870000e-004 | 1.554470e-004 |
| 500. | 1.889650e-004 | 2.129180e-004 | 1.678480e-004 |
| 600. | 1.980000e-004 | 2.150000e-004 | 1.933400e-004 |
| 700. | 1.986070e-004 | 2.271340e-004 | 1.801160e-004 |
| 800. | 2.400000e-004 | 2.460000e-004 | 2.305210e-004 |
| 1000. | 2.400000e-004 | 2.880000e-004 | 2.409300e-004 |

| Michaelis-Menten |  |  |  |
| --- | --- | --- | --- |
| Best-fit values |  |  |  |
| Vmax | 0.0004919 | 0.0005258 | 0.0004992 |
| Km | 940.5 | 853.0 | 1028 |
| Std. Error |  |  |  |
| Vmax | 8.546e-005 | 7.192e-005 | 9.866e-005 |
| Km | 274.0 | 202.6 | 330.4 |
| 95% Confidence Intervals |  |  |  |
| Vmax | 0.0002898 to 0.0006940 | 0.0003558 to 0.0006959 | 0.0002659 to 0.0007326 |
| Km | 292.4 to 1589 | 373.8 to 1332 | 246.4 to 1809 |
| Goodness of Fit |  |  |  |
| Degrees of Freedom | 7 | 7 | 7 |
| R square | 0.9669 | 0.9745 | 0.9647 |
| Absolute Sum of Squares | 1.355e-009 | 1.230e-009 | 1.433e-009 |
| Sy.x | 1.391e-005 | 1.325e-005 | 1.431e-005 |
| Constraints |  |  |  |
| Km | Km > 0.0 | Km > 0.0 | Km > 0.0 |
|  |  |  |  |
| Number of points |  |  |  |
| Analyzed | 9 | 9 | 9 |

**I86A with substrate L-pyroglutamyl-Phe-Leu-p-nitroanilide**

**E_T_** = 24 nM

| [Substrate in µM] | Enzyme Activity (V_0_ in OD_410nm_/Sec) | | |
| --- | --- | --- | --- |
| 100. | 6.690000e-005 | 6.573830e-005 | 8.321180e-005 |
| 200. | 8.100000e-005 | 8.800000e-005 | 9.615050e-005 |
| 250. |  | 1.180000e-004 | 1.023310e-004 |
| 300. | 1.260000e-004 | 1.200000e-004 | 1.197940e-004 |
| 350. | 1.290000e-004 | 1.320000e-004 | 1.259480e-004 |
| 400. | 1.360000e-004 | 1.300000e-004 | 1.345340e-004 |
| 450. | 1.441630e-004 | 1.439140e-004 |  |
| 500. | 1.480870e-004 | 1.390000e-004 | 1.466030e-004 |
| 550. | 1.599690e-004 |  | 1.720820e-004 |
| 600. | 1.609980e-004 | 1.680000e-004 | 1.770000e-004 |
| 700. | 1.734000e-004 |  | 1.563600e-004 |
| 800. | 1.795750e-004 | 1.879070e-004 | 1.992780e-004 |

| Michaelis-Menten |  |  |  |
| --- | --- | --- | --- |
| Best-fit values |  |  |  |
| Vmax | 0.0002572 | 0.0002583 | 0.0002598 |
| Km | 349.0 | 347.5 | 336.1 |
| Std. Error |  |  |  |
| Vmax | 1.509e-005 | 2.190e-005 | 3.096e-005 |
| Km | 48.29 | 65.06 | 94.15 |
| 95% Confidence Intervals |  |  |  |
| Vmax | 0.0002230 to 0.0002913 | 0.0002077 to 0.0003088 | 0.0001898 to 0.0003298 |
| Km | 239.8 to 458.3 | 197.4 to 497.5 | 123.1 to 549.0 |
| Goodness of Fit |  |  |  |
| Degrees of Freedom | 9 | 8 | 9 |
| R square | 0.9738 | 0.9518 | 0.8754 |
| Absolute Sum of Squares | 3.309e-010 | 5.401e-010 | 1.664e-009 |
| Sy.x | 6.064e-006 | 8.217e-006 | 1.360e-005 |
| Constraints |  |  |  |
| Km | Km > 0.0 | Km > 0.0 | Km > 0.0 |
|  |  |  |  |
| Number of points |  |  |  |
| Analyzed | 11 | 10 | 11 |

**Wild-type with substrate N-Benzoyl-Phe-Val-Arg- p-nitroanilide**

**E_T_ = 16nM**

Data sets:

| [Substrate in µM] | Enzyme Activity (V_0_ in OD_410nm_/Sec) | | |
| --- | --- | --- | --- |
| 10. | 7.710270e-005 | 6.251680e-005 | 6.797360e-005 |
| 20. | 1.348620e-004 | 1.361260e-004 | 1.254400e-004 |
| 30. | 1.796830e-004 | 1.993680e-004 | 1.867050e-004 |
| 40. | 2.233280e-004 | 2.630220e-004 | 2.505380e-004 |
| 50. | 2.985320e-004 | 2.949720e-004 | 2.811900e-004 |
| 80. | 3.366680e-004 | 3.037250e-004 | 3.322280e-004 |
| 120. | 3.857960e-004 | 3.796150e-004 | 3.586950e-004 |
| 180. | 4.310900e-004 | 4.312880e-004 | 4.191340e-004 |
| 260. | 4.989520e-004 | 5.218700e-004 | 5.016290e-004 |
| 400. | 4.822200e-004 | 4.669760e-004 | 4.772090e-004 |

| Michaelis-Menten |  |  |  |
| --- | --- | --- | --- |
| Best-fit values |  |  |  |
| Vmax | 0.0005798 | 0.0005706 | 0.0005693 |
| Km | 59.19 | 55.90 | 58.69 |
| Std. Error |  |  |  |
| Vmax | 2.091e-005 | 3.165e-005 | 2.391e-005 |
| Km | 6.174 | 9.131 | 7.148 |
| 95% Confidence Intervals |  |  |  |
| Vmax | 0.0005316 to 0.0006280 | 0.0004976 to 0.0006436 | 0.0005142 to 0.0006245 |
| Km | 44.95 to 73.42 | 34.85 to 76.96 | 42.20 to 75.17 |
| Goodness of Fit |  |  |  |
| Degrees of Freedom | 8 | 8 | 8 |
| R square | 0.9852 | 0.9638 | 0.9800 |
| Absolute Sum of Squares | 2.893e-009 | 7.069e-009 | 3.820e-009 |
| Sy.x | 1.902e-005 | 2.972e-005 | 2.185e-005 |
| Constraints |  |  |  |
| Km | Km > 0.0 | Km > 0.0 | Km > 0.0 |
|  |  |  |  |
| Number of points |  |  |  |
| Analyzed | 10 | 10 | 10 |

Substrate concentration in µM

**I86F with substrate N-Benzoyl-Phe-Val-Arg- p-nitroanilide**

**E_T_ = 31nM**

Data sets:

| [Substrate in µM] | Enzyme Activity (V_0_ in OD_410nm_/Sec) | | |
| --- | --- | --- | --- |
| 10. | 0.00019272 | 0.000156 | 0.000332518 |
| 30. | 0.000564 |  | 0.001134 |
| 50. | 0.000703174 | 0.000697 | 0.000329562 |
| 80. | 0.000979 | 0.000920211 |  |
| 100. | 0.001230 | 0.001130 | 0.001220 |
| 130. | 0.001290 | 0.000672 | 0.001440 |
| 150. | 0.001560 | 0.001574 | 0.001620 |
| 200. | 0.001810 | 0.001622 | 0.001730 |
| 250. | 0.002020 |  |  |
| 300. |  | 0.002120 | 0.002190 |
| 500. | 0.002650 | 0.002228691 | 0.002190 |
| 700. | 0.002547 | 0.002470 | 0.003224 |
| 1000. | 0.003536 | 0.002860 | 0.003480 |

| Michaelis-Menten |  |  |  |
| --- | --- | --- | --- |
| Best-fit values |  |  |  |
| Vmax | 0.003971 | 0.003420 | 0.004131 |
| Km | 243.2 | 232.3 | 257.8 |
| Std. Error |  |  |  |
| Vmax | 0.0002535 | 0.0003144 | 0.0005232 |
| Km | 37.12 | 52.78 | 79.67 |
| 95% Confidence Intervals |  |  |  |
| Vmax | 0.003406 to 0.004536 | 0.002709 to 0.004131 | 0.002948 to 0.005315 |
| Km | 160.5 to 325.9 | 112.9 to 351.7 | 77.59 to 438.0 |
| Goodness of Fit |  |  |  |
| Degrees of Freedom | 10 | 9 | 9 |
| R square | 0.9701 | 0.9387 | 0.9000 |
| Absolute Sum of Squares | 3.122e-007 | 4.495e-007 | 1.034e-006 |
| Sy.x | 0.0001767 | 0.0002235 | 0.0003390 |
| Constraints |  |  |  |
| Km | Km > 0.0 | Km > 0.0 | Km > 0.0 |
|  |  |  |  |
| Number of points |  |  |  |
| Analyzed | 12 | 11 | 11 |

**I86L with substrate N-Benzoyl-Phe-Val-Arg- p-nitroanilide**

**E_T_**= 24.7 nM

| [Substrate in µM] | Enzyme Activity (V_0_ in OD_410nm_/Sec) | | |
| --- | --- | --- | --- |
| 10. | 0.000152 | 0.000114 |  |
| 30. | 0.000294 | 0.000305 | 0.000337 |
| 40. | 0.000393 | 0.000472 | 0.000389 |
| 50. | 0.000499 | 0.000493 | 0.000468 |
| 60. | 0.000614 |  | 0.000560 |
| 70. | 0.000644 |  | 0.000640 |
| 80. |  | 0.000614 | 0.000667 |
| 90. |  | 0.000681 |  |
| 100. | 0.000761 | 0.000803 | 0.000756 |
| 130. | 0.000809 | 0.000847 | 0.000774 |
| 150. | 0.000800182 | 0.000864145 | 0.000859207 |
| 200. | 0.000860635 | 0.000961494 | 0.000959259 |
| 300. | 0.000924526 | 0.001060 | 0.000945215 |
| 400. | 0.001220 | 0.001060 | 0.001230 |
|  |  |  |  |
|  |  |  |  |

| Michaelis-Menten |  |  |  |
| --- | --- | --- | --- |
| Best-fit values |  |  |  |
| Vmax | 0.001321 | 0.001332 | 0.001398 |
| Km | 84.17 | 81.85 | 93.41 |
| Std. Error |  |  |  |
| Vmax | 8.846e-005 | 5.135e-005 | 7.625e-005 |
| Km | 14.56 | 8.349 | 12.44 |
| 95% Confidence Intervals |  |  |  |
| Vmax | 0.001124 to 0.001518 | 0.001218 to 0.001447 | 0.001228 to 0.001568 |
| Km | 51.72 to 116.6 | 63.25 to 100.5 | 65.70 to 121.1 |
| Goodness of Fit |  |  |  |
| Degrees of Freedom | 10 | 10 | 10 |
| R square | 0.9514 | 0.9841 | 0.9594 |
| Absolute Sum of Squares | 4.700e-008 | 1.569e-008 | 3.030e-008 |
| Sy.x | 6.856e-005 | 3.961e-005 | 5.505e-005 |
| Constraints |  |  |  |
| Km | Km > 0.0 | Km > 0.0 | Km > 0.0 |
|  |  |  |  |
| Number of points |  |  |  |
| Analyzed | 12 | 12 | 12 |

**I86A with substrate N-Benzoyl-Phe-Val-Arg- p-nitroanilide**

**E_T_** = 24nM

| [Substrate in µM] | Enzyme Activity (V_0_ in OD_410nm_/Sec) | | |
| --- | --- | --- | --- |
| 10. | 0.000114 |  |  |
| 30. | 0.000136 | 0.000137 | 0.000138196 |
| 50. | 0.000186 | 0.000181 |  |
| 70. | 0.000260 | 0.000296 | 0.00025504 |
| 100. | 0.000334 | 0.000337 | 0.000343768 |
| 130. | 0.000382 |  | 0.00038336 |
| 150. | 0.000408 | 0.000420 |  |
| 200. | 0.000469 |  | 0.000474649 |
| 220. | 0.000482 |  |  |
| 260. |  | 0.000540 |  |
| 300. |  | 0.000486 | 0.000442 |
| 500. | 0.000532 | 0.000522 | 0.000612 |
| 750. | 0.000608 | 0.000710 | 0.000607 |

| Michaelis-Menten |  |  |  |
| --- | --- | --- | --- |
| Best-fit values |  |  |  |
| Vmax | 0.0006851 | 0.0007560 | 0.0007071 |
| Km | 106.1 | 129.5 | 115.7 |
| Std. Error |  |  |  |
| Vmax | 3.077e-005 | 5.819e-005 | 4.240e-005 |
| Km | 13.27 | 28.26 | 20.75 |
| 95% Confidence Intervals |  |  |  |
| Vmax | 0.0006155 to 0.0007547 | 0.0006184 to 0.0008936 | 0.0006033 to 0.0008108 |
| Km | 76.10 to 136.1 | 62.66 to 196.3 | 64.95 to 166.5 |
| Goodness of Fit |  |  |  |
| Degrees of Freedom | 9 | 7 | 6 |
| R square | 0.9757 | 0.9424 | 0.9608 |
| Absolute Sum of Squares | 6.600e-009 | 1.555e-008 | 7.362e-009 |
| Sy.x | 2.708e-005 | 4.713e-005 | 3.503e-005 |
| Constraints |  |  |  |
| Km | Km > 0.0 | Km > 0.0 | Km > 0.0 |
|  |  |  |  |
| Number of points |  |  |  |
| Analyzed | 11 | 9 | 8 |
|  |  |  |  |

**Wild-type with substrate D-Val-Leu-Lys- p-nitroanilide**

**E_T_ = 31nM**

Data sets:

| [Substrate in µM] | Enzyme Activity (V_0_ in OD_410nm_/Sec) | | |
| --- | --- | --- | --- |
| 100. | 0.000025 | 0.0000295 | 0.00003193 |
| 150. |  | 0.000037 | 0.0000359852 |
| 200. | 0.000059 | 0.000044 | 0.0000441251 |
| 300. | 0.0000671 | 0.0000599 | 0.0000580571 |
| 400. | 0.0000855 | 0.0000796359 | 0.0000746956 |
| 500. | 0.000101 | 0.0000888606 | 0.0000887554 |
| 600. | 0.000102 | 0.0000898 | 0.0000889667 |
| 700. | 0.000109 | 0.0000937893 | 0.0000978964 |
| 800. | 0.00010926 | 0.000105 | 0.0000973224 |
| 1000. | 0.000132025 | 0.000113 | 0.000119902 |

| Michaelis-Menten |  |  |  |
| --- | --- | --- | --- |
| Best-fit values |  |  |  |
| Vmax | 0.0001935 | 0.0001746 | 0.0001789 |
| Km | 523.3 | 548.6 | 565.9 |
| Std. Error |  |  |  |
| Vmax | 1.697e-005 | 8.963e-006 | 1.570e-005 |
| Km | 96.91 | 57.21 | 99.64 |
| 95% Confidence Intervals |  |  |  |
| Vmax | 0.0001534 to 0.0002337 | 0.0001540 to 0.0001953 | 0.0001427 to 0.0002151 |
| Km | 294.1 to 752.5 | 416.7 to 680.6 | 336.1 to 795.7 |
| Goodness of Fit |  |  |  |
| Degrees of Freedom | 7 | 8 | 8 |
| R square | 0.9749 | 0.9909 | 0.9749 |
| Absolute Sum of Squares | 2.122e-010 | 7.067e-011 | 2.025e-010 |
| Sy.x | 5.506e-006 | 2.972e-006 | 5.031e-006 |
| Constraints |  |  |  |
| Km | Km > 0.0 | Km > 0.0 | Km > 0.0 |
|  |  |  |  |
| Number of points |  |  |  |
| Analyzed | 9 | 10 | 10 |

**I86L with substrate D-Val-Leu-Lys- p-nitroanilide**

**E_T_**=24.7 nM

| [Substrate in µM] | Enzyme Activity (V_0_ in OD_410nm_/Sec) | | |
| --- | --- | --- | --- |
| 10. | 7.300000e-005 | 1.100000e-004 | 9.418750e-005 |
| 40. | 1.900000e-004 | 1.920000e-004 | 2.242750e-004 |
| 60. | 2.720000e-004 | 2.510000e-004 | 2.761210e-004 |
| 100. | 3.960000e-004 | 4.300000e-004 | 3.836850e-004 |
| 120. | 5.050000e-004 | 5.020000e-004 | 4.786350e-004 |
| 150. | 5.170000e-004 | 5.300000e-004 | 5.273020e-004 |
| 200. | 5.370000e-004 | 5.440000e-004 | 5.315660e-004 |
| 250. | 5.930820e-004 | 6.059980e-004 | 5.818200e-004 |
| 300. | 6.282610e-004 | 6.301370e-004 | 6.369420e-004 |
| 400. | 6.451190e-004 | 6.527520e-004 | 6.440820e-004 |
| 550. | 7.597840e-004 | 7.750710e-004 | 7.695980e-004 |
| 650. | 6.590590e-004 | 7.318990e-004 | 6.986680e-004 |

| Michaelis-Menten |  |  |  |
| --- | --- | --- | --- |
| Best-fit values |  |  |  |
| Vmax | 0.0008385 | 0.0008851 | 0.0008580 |
| Km | 105.8 | 115.1 | 111.0 |
| Std. Error |  |  |  |
| Vmax | 3.995e-005 | 4.014e-005 | 3.359e-005 |
| Km | 15.72 | 15.70 | 13.28 |
| 95% Confidence Intervals |  |  |  |
| Vmax | 0.0007495 to 0.0009275 | 0.0007957 to 0.0009746 | 0.0007831 to 0.0009328 |
| Km | 70.80 to 140.8 | 80.11 to 150.1 | 81.41 to 140.6 |
| Goodness of Fit |  |  |  |
| Degrees of Freedom | 10 | 10 | 10 |
| R square | 0.9697 | 0.9735 | 0.9791 |
| Absolute Sum of Squares | 1.448e-008 | 1.317e-008 | 9.655e-009 |
| Sy.x | 3.805e-005 | 3.630e-005 | 3.107e-005 |
| Constraints |  |  |  |
| Km | Km > 0.0 | Km > 0.0 | Km > 0.0 |
|  |  |  |  |
| Number of points |  |  |  |
| Analyzed | 12 | 12 | 12 |

**Table S2**: Summary of kinetic experiments with L-pyroglutamyl-Phe-Leu-p-nitroanilide, N-Benzoyl-Phe-Val-Arg- p-nitroanilide and D-Val-Leu-Lys- p-nitroanilide.(kcat= Vmax / [E]_T_; extinction coefficient of liberated pNA is 8800 M^-1^ cm^-1^at 410nm).

|  | k_cat_ in S^-1^ | | | | Km in µM | | | | kcat/Km in M^-1^S^-1^ | | | |
| --- | --- | --- | --- | --- | --- | --- | --- | --- | --- | --- | --- | --- |
|  | Set1 | Set2 | Set3 | Mean ±SD | Set1 | Set2 | Set3 | Mean ±SD | Set1 | Set2 | Set3 | Mean ±SD |
| Substrate: L-pyroglutamyl-Phe-Leu-p-nitroanilide | | | | | | | | | | | | |
| WT | 2.91 | 2.96 | 2.87 | 2.91±0.05 | 266.00 | 258.00 | 260.70 | 261.57±4.07 | 10945.72 | 11478.54 | 11007.15 | 11143.8±291.51 |
| I86F | 2.78 | 2.95 | 2.67 | 2.80±0.14 | 91.63 | 102.60 | 90.19 | 94.81±6.79 | 30286.15 | 28777.31 | 29576.05 | 29546.5±754.85 |
| I86L | 2.26 | 2.42 | 2.30 | 2.33±0.08 | 940.50 | 853.00 | 1028.00 | 940.50±87.50 | 2406.24 | 2835.91 | 2234.10 | 2492.08±309.95 |
| I86A | 1.22 | 1.22 | 1.23 | 1.22±0.01 | 349.00 | 347.50 | 336.10 | 344.20±7.05 | 3489.41 | 3519.46 | 3659.96 | 3556.28±91.04 |
| Substrate: N-Benzoyl-Phe-Val-Arg- p-nitroanilide | | | | | | | | | | | | |
| WT | 4.12 | 4.05 | 4.04 | 4.07±0.04 | 59.19 | 55.90 | 58.69 | 57.93±1.77 | 69570.84 | 72496.54 | 68892.89 | 70320.09±1915.10 |
| I86F | 14.56 | 12.54 | 15.14 | 14.08 ±1.37 | 243.20 | 232.30 | 257.80 | 244.43±12.79 | 59853.83 | 53967.53 | 58739.18 | 57520.18±3126.76 |
| I86L | 6.08 | 6.13 | 6.43 | 6.21±0.19 | 84.17 | 81.85 | 93.41 | 86.48±6.12 | 72204.77 | 74869.67 | 68854.79 | 71976.41±3013.93 |
| I86A | 3.24 | 3.58 | 3.35 | 3.39±0.17 | 106.10 | 129.50 | 115.70 | 117.10±11.76 | 30573.47 | 27641.28 | 28937.0 | 29050.58±1469.39 |
| Substrate: D-Val-Leu-Lys- p-nitroanilide | | | | | | | | | | | | |
| WT | 0.71 | 0.64 | 0.66 | 0.67±0.04 | 523.30 | 548.60 | 565.90 | 545.93±21.42 | 1355.46 | 1166.659 | 1158.847 | 1226.99±111.33 |
| I86L | 3.86 | 4.07 | 3.95 | 3.96±0.11 | 105.80 | 115.10 | 111.00 | 110.63±4.66 | 36461.77 | 35378.34 | 35561.88 | 35800.66±579.85 |
